# Contralateral hip fractures and other osteoporosis-related fractures in hip fracture patients: incidence and risk factors. An observational cohort study of 1,229 patients

**DOI:** 10.1007/s00402-012-1520-9

**Published:** 2012-04-24

**Authors:** Anne J. H. Vochteloo, Boudewijn L. S. Borger van der Burg, Maarten A. Röling, Diederik H. van Leeuwen, Peter van den Berg, Arthur H. P. Niggebrugge, Mark R. de Vries, Wim E. Tuinebreijer, Rolf M. Bloem, Rob G. H. H. Nelissen, Peter Pilot

**Affiliations:** 1 Department of Orthopaedics, Reinier de Graaf Group, PO box 5011, 2600 GA Delft, The Netherlands; 2 Department of Orthopaedics, Leiden University Medical Centre, Leiden, The Netherlands; 3 Department of Surgery, Rijnland Hospital, Leiderdorp, The Netherlands; 4 Department of Surgery, Bronovo Hospital, The Hague, The Netherlands; 5 Department of Surgery, Reinier de Graaf Group, Delft, The Netherlands; 6 Department of Surgery-Traumatology Erasmus MC, University Medical Center, Rotterdam, The Netherlands

**Keywords:** Hip fracture, Contralateral, Bilateral, Osteoporosis, Risk factors

## Abstract

**Purpose:**

To report risk factors, 1-year and overall risk for a contralateral hip and other osteoporosis-related fractures in a hip fracture population.

**Methods:**

An observational study on 1,229 consecutive patients of 50 years and older, who sustained a hip fracture between January 2005 and June 2009. Fractures were scored retrospectively for 2005–2008 and prospectively for 2008–2009. Rates of a contralateral hip and other osteoporosis-related fractures were compared between patients with and without a history of a fracture. Previous fractures, gender, age and ASA classification were analysed as possible risk factors.

**Results:**

The absolute risk for a contralateral hip fracture was 13.8 %, for one or more osteoporosis-related fracture(s) 28.6 %. First-, second- and third-year risk for a second hip fracture was 2, 1 and 0 %. Median (IQR) interval between both hip fractures was 18.5 (26.6) months. One-year incidence of other fractures was 6 %. Only age was a risk factor for a contralateral hip fracture, hazard ratio (HR) 1.02 (1.006–1.042, *p* = 0.008). Patients with a history of a fracture (33.1 %) did not have a higher incidence of fractures during follow-up (16.7 %) than patients without fractures in their history (14 %). HR for a contralateral hip fracture for the fracture versus the non-fracture group was 1.29 (0.75–2.23, *p* = 0.360).

**Conclusion:**

The absolute risk of a contralateral hip fracture after a hip fracture is 13.8 %, the 1-year risk was 2 %, with a short interval between the 2 hip fractures. Age was a risk factor for sustaining a contralateral hip fracture; a fracture in history was not.

## Introduction

The incidence of osteoporosis has increased over the last decades in our aging population [1–3]. As advanced age and osteoporosis lead to enhanced bone fragility and increased fracture risk, the amount of osteoporosis-related fractures has also increased. Lifetime risk for developing an osteoporotic fracture is 30 % with an estimated amount of 9.0 million fractures worldwide in the year 2000 [4, 5]. Fractures of the proximal femur, distal radius, proximal humerus and vertebrae are the most frequently seen types of osteoporosis-related fractures. These fractures are related with increased morbidity and mortality. Hip fractures have the most devastating impact on a patients’ life with 1-year mortality rates of 18 up to 32 %, compared to 15 % after a vertebral fracture [2, 6–8]. Half of all hip fracture patients will never recover to their pre-fracture functional capacity and 25 % of these patients reside in a long-term care institution 1 year after sustaining a hip fracture [9–11].

Besides high mortality and high morbidity rates, an osteoporosis-related fracture has been identified as an important risk factor for sustaining subsequent fractures, particularly during the first 2 years after the initial fracture [12, 13]. The risk of sustaining a contralateral hip fracture within 2 years after the initial hip fracture is reported to be 4–10 % [14, 15]. As hip fractures are the most devastating fractures for patients, the main goal of this study was to assess the 1-year risk and absolute risk of sustaining a contralateral hip fracture in our cohort. Secondary, possible risk factors for sustaining a contralateral hip fracture were identified.

## Materials and methods

An observational cohort study of 1,229 consecutive hip fracture patients of 50 years and older, admitted to two teaching hospitals from January 2005 to July 2009. The study was retrospective for patients admitted between 2005 and 2008, and prospective for patients admitted between 2008 through June 2009. The first hip fracture sustained within this time frame was marked as the index hip fracture. Patients with a fracture due to a high-energy trauma or with a pathologic fracture were excluded. Osteoporosis-related fractures (contralateral hip, distal radius, proximal humerus and vertebrae at any level) in the history of all patients were retrospectively scored. All hospital databases (emergency department, clinical and radiological records and operating theatre database) were used to collect fracture data. All admissions of the patients were entered into our database. When a patient was not admitted to hospital, emergency room data were still entered into the database. By combining the digital files of emergency department admittance, hospital records and operating theatre data all potential hip and other fractures were scored as complete as possible.

From the hospital’s records, patient demographics like age, gender, ASA physical status classification, type of fracture, type of treatment, type of anaesthesia, were collected onto a case record form (CRF) [16].

Postoperative mortality has been documented by repeated consultation of the population registers present in every county in The Netherlands. For the assessment of concomitant fractures both before and after the index hip fracture, the patients record in the picture and archives system (PACS) was evaluated from January 2003 (2 years before the index fracture) up to January 2010. This time frame was chosen since 2003 a PACS was used in both hospitals. All low-energy trauma fractures of the contralateral hip, distal radius, proximal humerus and vertebrae at any level were scored. The patient record files were evaluated for notes on previous history for fractures occurred before 2003. If present, they were recorded at the CRF. As of 2008 all data were recorded prospectively at the CRF.

The 1-year incidence and prevalence of fractures of the contralateral hip, distal radius, proximal humerus and vertebrae both prior to and after the index hip fracture were determined.

Patients with bilateral hip fractures were compared to those with a unilateral hip fracture, regarding general demographics (age, gender, ASA classification, type of fracture and treatment), prevalence and 1-year incidence of concomitant fractures. This was done for patients with and without fractures prior to the index hip fracture. Finally, the different groups were compared regarding mortality rates.

It was not necessary to obtain approval from the local ethical committee due to the observational character of this surveillance study. Therefore, it is an evaluation of usual care as a part of good clinical practice. Since data could not be traced back to the individual patient there were no privacy issues.

### Statistical analysis

Categorical data are presented as the number of subjects in the category, along with the percentages. Chi-square tests and Fisher’s exact test were used for comparing groups of categorical data. All continuous data are presented as means with standard deviations (SD). The independent Student’s *t* test was used to compare groups of continuous data.

Fracture and mortality rates were expressed for different time periods calculated using the Kaplan–Meier method. Univariable and multivariable Cox regression analysis was used to calculate the hazard ratios and 95 % confidence interval (CI) to compare the difference in mortality and fracture risk in patients with or without previous fractures. In multivariable analysis the hazard ratios were adjusted for possible confounders: age, gender and general condition expressed as the ASA classification (I/II vs. III/IV).

Combining ASA I or II and III or IV classified patients in two groups was done as the separate groups of ASA I (*n* = 108) and ASA IV (*n* = 44) classified patients were too small to be analysed separately.

Age was categorized in three groups: 50–65, 65–85 years and older than 85 years.

*p* values lower than 0.05 were considered statistically significant. All data were analysed in SPSS 18.0 (SPSS Inc. Chicago, USA).

## Results

### Characteristics

1,229 hip fracture patients above 50 years were included, 891 female and 338 male. The median follow-up after the index hip fracture was 17.8 months [interquartile range (IQR) 28.6]. Mean (SD) age at admission for the index hip fracture was 81.7 (9.5) years. Women (mean 82.6, SD 9.0) were older than men (mean 79.4, SD 10.3, *p* < 0.001).

The majority of patients were treated with osteosynthesis (60.5 %), followed by (hemi-) arthroplasty (38.1 %). A small group was treated conservatively (1.4 %).

The overall 1-year mortality rate of the 1,229 patients was 23 % (95 % CI 21–26 %). More characteristics are shown in Table 1.

**Table 1:** Characteristics of unilateral and bilateral hip fracture patients and results of the univariable Cox regression analysis

	Study population *N* (%)	Unilateral hip fracture *N* (%)	Bilateral hip fracture *N* (%)	HR (CI)	*p* value
Median (IQR) follow-up in days	1,229 (100)	1,060 (86.2)	169 (13.8)		
	543 (873)	536 (886)	563 (810)		0.81*
Gender					
Female	891 (72.5)	768 (72.5)	123 (72.8)	1.15 (0.82–1.62)	0.41
Male	338 (27.5)	292 (27.5)	46 (27.2)		
Age, mean (SD)	81.7 (9.5)	81.7 (9.4)	82.0 (9.9)		0.74^#^
Age HR per year				1.02 (1.01–1.04)	0.011
Age HR per decade				1.25 (1.05–1.48)	0.011
ASA classification					
I–II	849 (69.1)	727 (68.6)	122 (72.2)	1.07 (0.76–1.49)	0.71
III–IV	380 (30.9)	333 (31.4)	47 (27.8)		
Fracture type					
Neck of femur	704 (57)	617 (58)	87 (51.5)		
(Inter) trochanteric	485 (39)	412 (39)	73 (43.2)		
Subtrochanteric	40 (3)	31 (3)	9 (5.3)		0.11
Anaesthesia					
Spinal	1,129 (92)	1,032 (92)	97 (91)		
General	83 (7)	75 (7)	8 (7)		0.94

Female gender and ASA I–II are reference categories

*HR* Hazard ratio, *CI* confidence interval, *IQR* interquartile range, *ASA* American society of anesthesiologists physical status classification

* Mann–Whitney test

^#^ *t* test

### Bilateral hip fractures

169 of the 1,229 patients had bilateral hip fractures, indicating an absolute risk of 13.8 %. The first-, second-, and third-year risk of a contralateral hip fracture was 2, 1, and 0 %, respectively (Table 2).

**Table 2:** Absolute risk and risk per year after the index fracture (both in %) for a subsequent (hip) fracture and for mortality during follow-up

	Absolute risk	Year 1	Year 2	Year 3	Year 4
Subsequent fracture risk	28.6	6	3	1	1
Subsequent hip fracture risk	13.8	2	1	0	0
Mortality risk	36.0	23	11	10	11

Of the 169 patients with bilateral hip fractures, 115 sustained a hip fracture before the index hip fracture, i.e., the index fracture was their second hip fracture. In 54 patients the second hip fracture occurred during follow-up after the index fracture, i.e., the index fracture was the first hip fracture. The median (IQR) interval between the two hip fractures in all 169 patients was 18.5 months (26.6), 36.1 % occurred with 1 year and 61.5 % was sustained in 2 years.

The median (IQR) interval between both hip fractures in the 54 patients that suffered a contralateral hip fracture during follow-up was 231 (434) days. As the follow-up of this group was too short to calculate reliable fracture incidence ratios, further analysis was performed in the entire population of 169 patients.

The mean (SD) age of patients at admission for their first hip fracture in the bilateral hip fractures group was 75.9 (11.5) years. This was lower than the mean (SD) age 81.7 (9.4) years, of the 1,060 unilateral hip fracture patients (*p* < 0.001). The mean (SD) age at time of the second hip fracture was 82.0 (9.9) years, not different from the unilateral fracture group. The male to female ratio, the mean age, type of fracture and type of anaesthesia in the unilateral group were equal to the bilateral hip fracture group (Table 1).

In univariable Cox regression analysis, the hazard ratio for men versus women to sustain a second hip fracture was 1.15 (0.82–1.62, *p* = 0.41), for ASA III–IV versus I–II 1.07 (0.76–1.49, *p* = 0.71), for age per year 1.02 (1.01–1.04, *p* = 0.011) and age per decade 1.25 (1.05–1.48, *p* = 0.011).

In multivariable Cox regression analysis, the hazard ratio for men versus women for sustaining a contralateral hip fracture was 1.24 (0.88–1.75, *p* = 0.22), for ASA III–IV versus I–II 0.98 (0.69–1.38, *p* = 0.90), for age per year 1.02 (1.01–1.04, *p* = 0.008) and age per decade 1.27 (1.06–1.51, *p* = 0.008).

### Concomitant osteoporosis-related fractures and risk factors

In 407 patients (33.1 %) an osteoporosis-related fracture prior to the index hip fracture was found. This group of 407 was compared to the 821 patients who did not suffer fractures prior to the index event.

The absolute risk of a contralateral hip fracture during follow-up in the group with fractures was 5.1 % (*n* = 21) and in the group without fractures 4.0 % (*n* = 33). The 1-year risk for a contralateral hip fracture for the prior fracture versus the non-prior fracture group was 3.0 versus 2.0 %. In univariable Cox regression analysis the hazard ratio for sustaining a contralateral hip fracture for the fracture versus the non-fracture group was 1.29 (0.75–2.23, *p* = 0.36).

The absolute risk of sustaining an osteoporosis-related fracture in the population without fractures in their medical history was 14.0 % with a 1-year risk of 9 %. In the group that did sustain prior fractures, the absolute risk was 16.7 % and the 1-year risk 9 %. In univariable Cox regression analysis the hazard ratio for sustaining an osteoporosis-related fracture in the population with fractures in their medical history versus the population without fractures in their medical history was 1.19 (0.88–1.61, *p* = 0.25).

The risks of sustaining different osteoporosis-related fractures after previous sustained fractures are listed in Table 3. A previous fracture was only a significant risk factor for sustaining a distal radius fracture (HR 1.66, 1.07–2.59, *p* = 0.025).

**Table 3:** One-year risks (in %) for different osteoporosis-related fractures for the population with and without previous fractures

With previous fractures				Without previous fractures			HR (CI)	*p* value
	*N*	%	One-year risk	*N*	%	One-year risk		
Hip	21	5.1	3.0	33	4.0	2.0	1.29 (0.75–2.23)	0.36
Distal radius	36	8.8	2.0	43	5.2	2.0	1.66 (1.07–2.59)	0.025
Humerus	20	4.9	2.0	32	3.9	1.0	1.27 (0.72–2.21)	0.41
Vertebrae	42	10.3	2.0	69	5.0	3.0	1.22 (0.83–1.79)	0.32

*HR* hazard ratio, *CI* confidence interval

The 1-year risk after the index fracture for osteoporosis-related fractures was 9 % in both women and men and 4 % in both men and women in the second year. In univariable Cox regression analysis, the hazard ratio for sustaining an osteoporosis-related fracture for male gender versus female was 0.95 (0.68–1.31, *p* = 0.74).

The absolute risk of sustaining osteoporosis-related fractures after the index hip fracture was 15.9 % for ASA I/II and 12.6 % for ASA III/IV classified patients. The 1-year risk of osteoporotic fractures was 9 % for ASA I/II versus 8 % in ASA III/IV. In univariable Cox regression analysis the hazard ratio for sustaining an osteoporosis-related fracture for ASA III–IV versus ASA I–II was 0.79 (0.57–1.09, *p* = 0.15).

No differences were seen in fracture risks in different age categories. More characteristics of the different age groups are shown in Table 4.

**Table 4:** Fractures in history and one-year (hip) fracture risk for three age categories

Age (years)	*N* (% of total population)	Fractures in history *N* (%)	One-year contralateral hip fracture risk (%)	HR (CI)	*p* value	One-year fracture risk (%)	HR (CI)	*p* value
50–65	87 (7.1)	23 (5.6)	3			11		
65–85	633 (51.5)	206 (50.5)	2	0.57 (0.23–1.39)	0.21	9	0.97 (0.56–1.70)	0.92
>85	509 (41.4)	179 (43.9)	3	0.65 (0.27–1.60)	0.35	8	0.85 (0.48–1.51)	0.57

Age group 50–65 was used as reference category

*HR* hazard ratio, *CI* confidence interval

### Mortality

The 1-year mortality risk and hazard ratios for mortality for the age categories, gender, ASA classification, the occurrence of a bilateral hip fracture, a fracture in the history, a fracture during follow-up and a hip fracture during follow-up are presented in Table 5. The hazard ratios for mortality were significantly higher for the age categories 65–85 years and older than 85 years, male gender and ASA III–IV, but not for the occurrence of a bilateral hip fracture, a fracture in the history and not for having a (hip) fracture during follow-up compared with the reference categories.

**Table 5:** Mortality for age categories, gender, ASA classification, for patients with or without bilateral hip fractures, a fracture in the history, a fracture during follow-up and a hip fracture during follow-up

	*N* (% of population)	One-year mortality (%)	HR (CI) for mortality	*p* value
Age categories (years)				
50–65	87 (7.1)	3		
65–85	633 (51.5)	20	5.24 (2.33–11.81)	<0.001
>85	509 (41.4)	33	9.10 (4.05–20.47)	<0.001
Gender				
Female	891 (72.5)	23		
Male	338 (27.5)	26	1.23 (1.00–1.51)	0.046
ASA				
I–II	849 (69.1)	20		
III–IV	380 (30.9)	33	1.94 (1.61–2.35)	<0.001
Bilateral hip fracture				
No	1,060 (86.2)	25		
Yes	169 (13.8)	19	0.76 (0.57–1.03)	0.073
Fracture in history				
No	821 (66.8)	24		
Yes	408 (33.2)	24	0.88 (0.72–1.08)	0.22
Fracture during FU				
No	1,046 (85.1)	25		
Yes	183 (14.9)	21	0.91 (0.70–1.18)	0.47
Hip fracture during FU				
No	1,175 (95.6)	24		
Yes	54 (4.4)	19	0.87 (0.55–1.38)	0.56

Age 50–65, female gender and ASA I–II are reference categories

*HR* hazard ratio, *CI* confidence interval, *FU* follow-up, *ASA* American Society of Anesthesiologists physical status classification

## Discussion

Our main goal was to describe the 1-year risk and absolute risks of sustaining a subsequent second hip fracture and other osteoporosis-related fractures in a hip fracture cohort. The absolute risk of a contralateral hip fracture was 13.8 %, the 1 year risk 2 %, for the other osteoporosis-related fractures this was 28.6 and 6 %, respectively. The median interval between the first and the second hip fracture was rather short, 18.5 months. The risk of a new osteoporosis-related fracture was not higher in patients with a fracture in their medical history than in those that did not.

Our second goal was to describe independent risk factors for sustaining a subsequent second hip fracture. We compared the population with a bilateral hip fracture with the unilateral population. There was no difference between patient’s gender distribution, ASA classification, and type of fracture or mortality rate between the uni- and bilateral hip fractures population. A higher age was the only risk factor that could be identified for sustaining a contralateral hip fracture.

Providing reliable concomitant fracture incidence rates and identifying risk factors in hip fracture patients can help in decision-making policies for fracture prevention, like osteoporosis prophylaxis. Furthermore, more accurate information about the future can be provided to both patient and family with respect to be expected fractures.

In our population, the 1-year risk of a second hip fracture was 2.0 %. Lawrence et al. [17] found in their recent study of a large cohort of 6,331 patients a comparable 1 year incidence of 2.7 %. Other prospective studies found the same incidence of around 2 % [18, 19]. However, Lönnroos et al. [14] found a higher contralateral hip fracture incidence of 5 %. The fact that they analysed a smaller population (501 patients) might account for the difference. Overall, 28.6 % of our patients had one or more osteoporosis-related fractures in our cohort. This resembles the lifetime risk for developing an osteoporotic fracture as reported by Klotzbuecher and Cummings [2, 4]. Our 1- and 2-year risk for other osteoporosis-related fractures were 6 and 3 %. This resembles the figures reported by van Helden et al. [13] who found a cumulative incidence of 10.8 % after 2 years follow-up. Although female gender is described as a risk factor in many studies, our cohort did not support this suggestion [3, 9, 20]. Rates of successive fractures after admission for a hip fracture were also not significantly higher in patients with prior fractures compared to those without. We found that a previous fracture was only a risk factor for sustaining a distal radius fracture in the future, not for other types of fractures. This is contrary to other series that reported a previous fracture at any site to be a significant risk factor for a future fracture. This might be explained by our retrospective collecting of data of previous fractures, accounting for a possible loss of fractures. A limitation is the potential loss of fractures in the retrospective review of medical charts. This might have led to an underestimation of fracture rates, thus influencing the calculated risk factors.

A multivariable Cox regression analysis on risk factors for a contralateral hip fracture showed only a significant influence of age, not of gender and ASA classification. This is an expected outcome; the more years lived, the more risk to sustain a fracture.

Patient’s characteristics of the entire population and the bilateral hip fracture population were comparable. However, patients admitted for a second hip fracture did sustain their first hip fracture on a significant lower age than patients admitted for their first hip fracture (75.9 vs. 81.7 years, *p* < 0.001). Therefore, it might be good practice to screen patients who sustain their first hip fracture at a younger age thoroughly for osteoporosis and other risk factors.

The median (IQR) interval between the two hip fractures in all 169 patients was 18.5 months (26.6), 36.1 % occurred with 1 year and 61.5 % was sustained in 2 years. Nymark [21] (9,990 patients) showed that 50 % of the contralateral hip fractures occurred within 12 months in men, and within 19 months in women. Other reported mean intervals between two hip fractures differ from 2.1 years (Chevally, 4,115 patients), 2.3 years (Kok, 1,604 patients), 3.3 years (Schroder, 3,898 patients) to 4.3 years (Fukushima 835 patients) [22–25]. The latter reported more that 70 % of all contralateral hip fractures to occur within 5 years, resembling our findings [25]. Therefore, the interval between two hip fractures is relatively short. Therefore, the interval between two hip fractures is relatively short. The effectiveness of osteoporosis medication is high, with relatively early results; commonly used osteoporosis agents like risedronate and alendronate significantly reduce the incidence of non-vertebral fractures (21–39 %) compared to placebo during 3 or more years of follow-up [26–31]. In post hoc analyses of these trial data, the reduction of non-vertebral fractures was present at 6 months for 5 mg daily dosing of risedronate [32] and at 12 months for 10 mg daily dosing of alendronate [33] or 24 months for 5 mg daily dosing of alendronate [26, 34].

These findings emphasize the importance of early screening for osteoporosis after a fracture; starting osteoporosis medication can prevent subsequent fractures in the future.

We presented a large series on hip fracture patients with a median follow-up of 2 years. The main limitation of our study was the retrospective collection of a part of the fracture patient data. This might potentially have led to an underestimation of the incidence of fracture from the medical chart. Although in this retrospective part of the study we vigorously analysed all radiographs and patient charts for presence of fractures after the index hip fracture, thus minimizing this potential error of underestimating fracture incidence. Furthermore, the reported fracture rates are comparable to a recent Dutch study [13]; therefore, the level of underestimation of the incidence of fractures might be low. Another limitation is the lack of data on bone mineral density; the actual number of patients suffering from osteoporosis is unknown. Finally, no data on the start of osteoporosis medication after the index fracture were available. This could have influenced the fracture rates during follow-up.

In conclusion, this large series adds important information to existing literature on hip fracture incidence rates and identifies risk factors. It emphasizes the importance of osteoporosis screening and treatment to prevent subsequent fractures because of the good and early effectiveness of current osteoporosis medication. Our outcomes can be used as a baseline for evaluating the efficacy of present osteoporosis screening and treatment modalities for successive fracture rates.
